# Safety and efficacy of oxycodone for refractory dyspnea in end-stage heart failure patients with chronic kidney disease: a case series of eight patients

**DOI:** 10.1186/s40780-024-00384-4

**Published:** 2024-10-07

**Authors:** Masayuki Tanaka, Hirofumi Maeba, Takeshi Senoo, Nana Yoshimiya, Haruna Ozaki, Kazuki Uchitani, Noboru Tanigawa, Kazuichi Okazaki

**Affiliations:** 1 Faculty of Pharmaceutical Sciences, Setsunan University, 45-1 Nagaotoge-Cho, Hirakata, Osaka 573-0101 Japan; 2 Department of Pharmacy, Kansai Medical University Hospital, Hirakata, Japan; 3 Division of Cardiology, Department of Medicine II, Kansai Medical University, Hirakata, Japan; 4 Maeba Clinic, Ibaraki, Japan; 5 Senoo Naika, Hirakata, Japan; 6 Department of Nursing, Kansai Medical University Hospital, Hirakata, Japan; 7 Department of Internal Medicine, Kansai Medical University Kori Hospital, Neyagawa, Japan

**Keywords:** Chronic kidney disease, End-stage heart failure, Morphine, Oxycodone, Refractory dyspnea

## Abstract

**Background:**

Morphine is effective in palliative care for patients with end-stage heart failure; however, its use is avoided in patients with impaired renal function because it tends to induce adverse effects. Although oxycodone has been reported to be a useful alternative, the evidence is insufficient. Therefore, we investigated the safety and efficacy of oxycodone in eight patients with end-stage heart failure complicated by chronic kidney disease.

**Methods:**

This single-center retrospective study reviewed patients with end-stage heart failure who were referred to the heart failure multidisciplinary team at our institution and administered oxycodone for refractory dyspnea during hospitalization between January 2011 and December 2018. We examined the details of oxycodone usage, vital signs, and the Modified Borg Scale (MBS), which quantifies the symptoms of dyspnea and adverse events.

**Results:**

Oxycodone was administered for refractory dyspnea in eight patients with end-stage heart failure [mean age: 81 years, men: 4, New York Heart Association functional class IV: 8, median left ventricular ejection fraction: < 40% (*n* = 6) and ≥ 50% (*n* = 2)]. Renal function was reduced in all patients; the estimated glomerular filtration rate (eGFR) in seven patients was < 30 mL/min/1.73 m^2^. The median initial intravenous dose of oxycodone was 7.05 mg/day (range: 5–10 mg/day), and the average duration of administration was 15.8 days. Significant decreases in MBS (before: median 9, range 7–10 vs. after: median 2.5, range 1–8; *p* < 0.01) were observed at a median of 2.0 days (range: 2 h to 7 days) after beginning oxycodone administration. Systolic blood pressure, heart rate, and respiratory rate were not significantly altered after treatment. Adverse events, including constipation, nausea, and tremors, were observed in three patients. However, no lethal adverse events related to oxycodone treatment occurred during treatment.

**Conclusions:**

This study revealed the clinical practice of oxycodone treatment and suggested that it is an alternative therapy as a viable palliative for refractory dyspnea in patients with end-stage heart failure who should avoid the use of morphine.

## Background

The major symptoms of end-stage heart failure are dyspnea, general fatigue, pain, anorexia, and depression. Some studies on end-stage heart failure have reported frequent occurrences of dyspnea, general fatigue, and pain (60–88%, 69–82%, and 35–78%, respectively) [1–3]. These symptoms may stem from physiological dysfunction associated with low cardiac output, including pulmonary congestion, fluid retention, and cardiac insufficiency, as well as from anxiety, anger, pain, and depression related to the intensity of dyspnea [4–7]. If a patient continues to experience unbearable distress following conventional therapy for dyspnea, treatment with opioids should be considered palliative care. The European and Japanese heart failure guidelines suggest morphine treatment for refractory dyspnea as palliative care in patients with end-stage heart failure [8], based on previous studies that reported the effectiveness of morphine administration for dyspnea in patients with chronic heart failure [9–11]. However, morphine treatment may be avoided in cases of renal failure due to serious adverse effects, such as respiratory depression caused by the accumulation of active metabolites of morphine. In these cases, oxycodone, which is less likely to accumulate during renal failure, may be an alternative to morphine. We previously reported a case where oxycodone improved dyspnea without adverse effects in a patient with end-stage heart failure and renal dysfunction [12]. However, the safety and efficacy of oxycodone have not been fully established. Accordingly, we aimed to investigate the safety and efficacy of oxycodone in eight patients with end-stage heart failure and renal dysfunction at our institution.

## Methods

### Study design and population

This single-center retrospective study was conducted by members of the heart failure multidisciplinary team at Kansai Medical University Hospital. This study was approved by the ethics review board of our institution (approval number 2018168). We retrospectively reviewed patients who were hospitalized at our institution and referred to the heart failure multidisciplinary team between January 2011 and December 2018. Patients with end-stage heart failure who were hospitalized for heart failure and received oxycodone as palliative care for refractory dyspnea were included in this analysis. The attending doctors finally decided to administer oxycodone to patients with end-stage heart failure who were experiencing intractable severe symptoms unresponsive to ordinary palliative care approaches, considering the preferences of the patients and their families. A multidisciplinary team and the attending doctors determined the administration route based on the patient’s condition and preferences. The oxycodone dose was determined on a case-by-case basis, considering patients’ age, physical constitution, renal function, and symptom severity. Patients with comorbid cancers were excluded.

### Definition and measurements

This retrospective study was based on a review of the medical charts. We investigated the detailed data on oxycodone usage, including administration route, dose, and duration. Temporal changes in vital signs, including respiratory rate, blood pressure, and heart rate, were obtained. We evaluated the efficacy of oxycodone treatment using the Modified Borg Scale (MBS), which quantifies dyspnea symptoms on a scale of 0 (no symptoms) to 10 (worst possible symptoms). The MBS enables the assessment of the respiratory discomfort perceived by the patient [12, 13] (Table 1). MBS scores were collected once every 30 min to 2 h following the initiation of oxycodone treatment and subsequently 1 to 2 times per day based on meal times and nurse shift changes. The data were obtained directly from patients.

**Table 1:** Modified borg scale

0	No Breathlessness
0.5	Very very slight (just noticeable)
1	Very slight
2	Slight Breathlessness
3	Moderate
4	Somewhat severe
5	Severe breathlessness
6	
7	Very severe breathlessness
8	
9	Very very severe (almost maximum)
10	Maximum

The modified Borg Scale score that documented the patient’s respiratory discomfort was recorded based on the current level of the patient’s subjective evaluation or the records of nurses involved in his treatment during his stay in the coronary care unit

 Vital signs and symptom scale data were collected both before initiating treatment and when the MBS showed the most improvement following the administration of oxycodone. If oxycodone was discontinued, the reasons for discontinuation were further investigated. Common adverse events of oxycodone, such as nausea, vomiting, constipation, delirium, and tremors, were evaluated from medical chart descriptions.

### Statistics

The Wilcoxon signed-rank test was used to test for differences in MBS, systolic blood pressure, heart rate, and respiratory rate before and after oxycodone treatment. All tests were two-tailed, and statistical significance was set at *p* < 0.05. All analyses were performed using JMP Pro version 17.2.0 (SAS Institute, Cary, NC, USA).

## Results

### Baseline characteristics of patients with heart failure who received oxycodone treatment

During the study period, oxycodone was administered to eight patients with end-stage heart failure for refractory dyspnea at our institution. The baseline patient characteristics and medications administered before oxycodone treatment are presented in Table 2.

**Table 2:** Baseline clinical characteristics

Case	Sex	Age	Heart failure cause disease	BMI (kg/m 2 )	eGFR (mL/min/1.73 m 2 )	LVEF (%)	SPO_2 _(%)	Medications						
								β-blocker	ACEI/ARB	MRA	Loop diuretic	Tolvaptan	Inotropic agent	Anti coagulants
1	F	75	DCM, AF	16.7	12	15	96	+		+	+	+	+	Warfarin
2	M	67	DCM, AF	21.6	26	26	96	+		+	+	+	+	Rivaroxaban
3	F	84	IHD, AS	18.4	16	74	99				+	+		
4	M	95	IHD	24.4	13	61	97	+			+		+	
5	F	81	AF, MR	24.5	17	23	99	+	+	+	+		+	Warfarin
6	M	77	ICM	17.9	41	22	97	+		+	+		+	
7	M	74	DCM, AF	21.4	29	38	96	+	+		+		+	Warfarin
8	F	88	ICM	24.8	14	33	93	+			+			

*DCM* Dilated cardiomyopathy, *ICM* Idiopathic cardiomyopathy, *IHD* Ischemic heart disease, *AF* Atrial fibrillation, *AS* Aortic stenosis, *MR* Mitral regurgitation, *LVEF* Left ventricular ejection fraction, *ACEI* Angiotensin-converting enzyme inhibitor, *ARB* Angiotensin receptor blocker, *MRA* Mineralocorticoid receptor antagonist

The mean age was 81 years, and four patients were male (50%). All patients were classified as New York Heart Association functional class IV, corresponding to ACCF/AHA stage D, and the left ventricular ejection fractions were < 40% (*n* = 6) and ≥ 50% (*n* = 2). The etiology of heart failure was dilated cardiomyopathy in five cases, ischemia in two cases, and valvular disease in two cases; four of these cases had chronic atrial fibrillation and were on concomitant anticoagulants. Renal function was reduced in all patients; the estimated glomerular filtration rate (eGFR) in seven patients was < 30 mL/min/1.73 m^2^. In all eight cases, loop diuretics were administered, and beta-blockers were also administered in seven cases. An angiotensin-converting enzyme (ACE) inhibitor or angiotensin receptor blocker (ARB) was administered in two cases. In Cases 1–4 and 8, ACE inhibitors or ARBs were discontinued before oxycodone injection, given the decline in renal function and difficulty in maintaining blood pressure. During oxycodone administration, no adjustments were made to the oral medications for Cases 1–3 and 7, and these medications were continued as prescribed. In contrast, in Cases 4, 6, and 8, all oral medications were discontinued after 2, 29, and 2 days of oxycodone initiation, respectively, to prevent aspiration due to the addition of sedatives. In Case 5, warfarin was reduced on the 4th day of oxycodone administration due to a significant increase in PT-INR, but no changes were made to the other medications. Additionally, for Cases 1, 2, and 4–7, in which cardiotonic drugs were administered, no dose increases were made during oxycodone treatment. Specifically, in Case 1, due to an improvement in dyspnea on the 11th day of oxycodone administration, the dose of the cardiotonic drug (dobutamine) was reduced from 7.5γ to 6.5γ, with no recurrence of dyspnea. Oxycodone treatment was discontinued the following day.

### Feasibility of oxycodone treatment for refractory dyspnea in patients with end-stage heart failure

The vital signs at baseline and after treatment for each patient are presented in Table 3.

**Table 3:** Characteristics before and after oxycodone administration

Case	Starting dosage (mg/day)	Maintenance dosage (mg/day)	Elapsed time until MBS improved well	MBS score		sBP (mmHg)		Heart rate (bpm)		Respiratory rate (bpm)		Adverse effect	Period of administration (day)	Reasons for termination
				Before	After	Before	After	Before	After	Before	After			
1	10	10	2 days	10	2	111	94	86	100	25	16	Constipation, nausea, tremor	12 days	Improved
2	10	10	7 days	10	2	80	100	110	90	15	20	None	29 days	Improved
3	6.6	10	2 days	9	8	98	113	103	108	19	26	None	4 days	Death
4	5	10	2 days	10	7	111	102	80	54	23	16	None	6 days	Death
5	5	5	1 day	8	3	85	80	133	102	18	18	PT-INR prolonged, tremor	10 days	Improved
6	7.5	7.5	1 day	10	2	102	111	95	78	26	20	None	35 days	Death
7	6	6	2 h	7	1	99	107	94	92	22	18	Tremor	8 days	Improved
8	10	10	2 days	7	3	129	115	71	72	23	20	None	23 days	Death

*MBS* Modified Borg scale, *sBP* Systolic blood pressure, *PT-INR* International normalized ratio of prothrombin time, *Before* Before oxycodone administration, *After* When MBS scores had improved

**Fig. 1:**
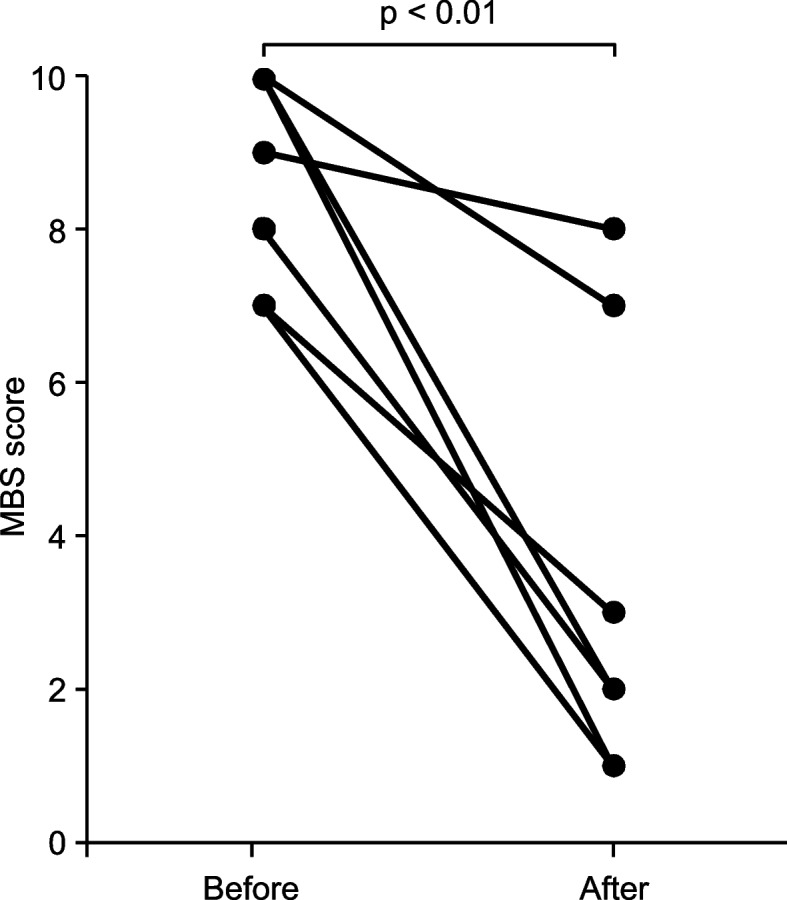
MBS scores before and after oxycodone administration

The median starting dose of oxycodone was 7.05 mg (range: 5–10 mg), and the median maintenance dose was 10 mg (range: 5–10 mg), with increases in Cases 3 and 4. The median elapsed time until MBS improvement was 2.0 days (range: 2 h to 7 days), and seven of the eight cases showed MBS improvement within 2 days. A significant reduction was observed in MBS (before: median 9, range 7–10 vs. after: median 2.5, range 1–8; *p* < 0.01) after starting oxycodone administration, as shown in Fig. 1.

**Fig. 2:**
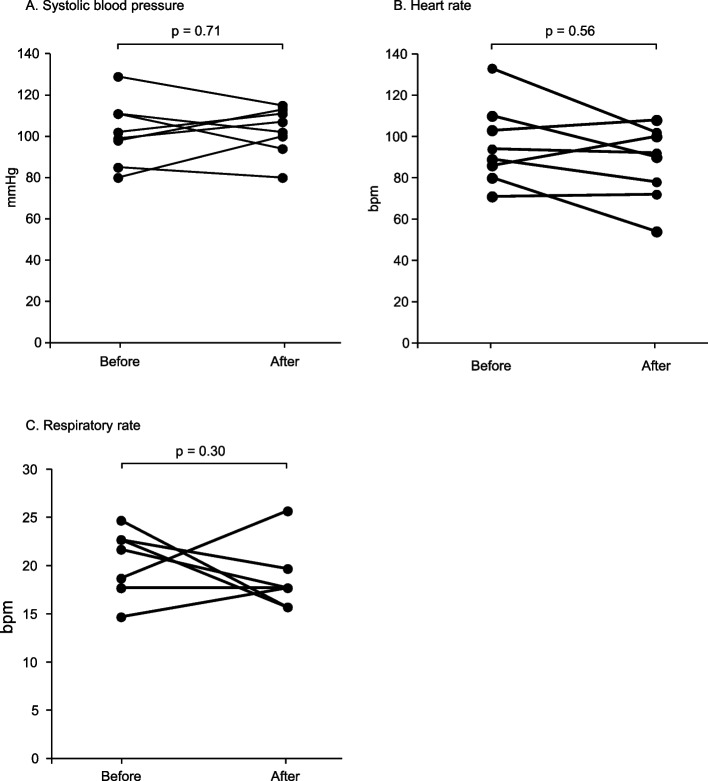
Vital signs before and after oxycodone administration. **A** Systolic blood pressure, **B** heart rate (bpm), **C** respiratory rate (breaths per minute)

Other vital signs, including systolic blood pressure, heart rate, and respiratory rate, were not significantly altered before and after starting oxycodone treatment (Fig. 2).

### Adverse events of oxycodone treatment in patients with end-stage heart failure

Oxycodone-induced adverse effects, including constipation, nausea, tremor, and prolonged international normalized ratio of prothrombin time (PT-INR) (in one patient using warfarin), were observed in three patients but were adequately managed with dose adjustment and symptomatic treatment. Constipation was treated with sennoside or lubiprostone, and alternatively, sorbitol solution and lactulose syrup were also effective. The nausea was treated with metoclopramide. The tremors observed in three patients (Cases 1, 5, and 7) were managed by decreasing the oxycodone dosage without precipitating dyspnea. In Case 5, the PT-INR increased from 2.0 to 2.6 after oxycodone treatment, requiring a reduction in the warfarin dose without bleeding.

### Outcome of patients with oxycodone administration

Dyspnea in four patients was completely relieved, and oxycodone was eventually discontinued. However, Case 4 experienced fatigue even after a stepwise increase in oxycodone, and midazolam was added on day 4. Similarly, Case 8 reported fatigue despite improvement in dyspnea, and midazolam was administered on day 3. In both cases, fatigue was alleviated after midazolam administration. Finally, four patients (Cases 3, 4, 6, and 8) died from a lack of response to treatment with inotropic agents and diuretics, resulting in an exacerbation of heart failure itself, regardless of the adverse effects of oxycodone. However, dyspnea in these four patients improved at the beginning of their treatment with oxycodone. The mean duration of oxycodone in the symptom improvement and death groups was 14.8 [8, 29] and 17 [4, 35] days, respectively, with no significant differences (Table 3).

## Discussion

In this retrospective survey of oxycodone treatment for refractory dyspnea as palliative care in real-world heart failure practice, we found the following results. First, oxycodone treatment significantly decreased MBS without affecting respiratory rate, heart rate, or blood pressure. Second, adverse effects due to oxycodone were effectively managed with dosage adjustment and symptomatic treatment without inducing serious adverse effects, such as respiratory depression. This study’s findings indicate that oxycodone may be a new treatment option for patients with end-stage heart failure in real-world practice.

### Dose and timing of oxycodone treatment in end-stage heart failure practice

The efficacy of morphine for dyspnea in patients with end-stage heart failure has been reported, with its effect at smaller doses than those for pain control [9, 10, 14]. Although no consensus on the dosage exists for heart failure, symptomatic relief has been reported with morphine tablets 5 mg four times a day (morphine injection equivalent 10 mg/day) [10]. In the field of heart failure, there are numerous reports on the introduction of morphine at 5–10 mg/day or smaller doses by continuous intravenous or subcutaneous infusion, similar to that in the field of oncology. However, caution should be exercised in patients with impaired renal function (creatinine clearance should be less than 30 mL/min), as the accumulation of morphine-3-glucuronide and 6-glucuronide, the active metabolites of morphine, may cause hypersedation, delirium, and respiratory depression [15, 16]. However, oxycodone, similar to morphine, has been reported to be useful for dyspnea in oncology [16, 17]. It can be used relatively safely even in cases of impaired renal function (as a rule of thumb, creatinine clearance of 10 mL/min or more) and can be administered to patients who have difficulty receiving morphine [18]. In heart failure, continuous intravenous or subcutaneous administration of oxycodone 4.8–10 mg/day has been reported to improve dyspnea, and it can be safely administered to patients with severe renal dysfunction [12, 19]. In this study, induction was conducted at 5–10 mg/day, and its efficacy was confirmed. Oxycodone should be carefully considered as an alternative drug in cases where morphine is difficult to use, such as in patients with impaired renal function who develop delirium. The method for initiating opioids for dyspnea is detailed in the “Approaches to Managing Pharmacotherapy in Palliative Care for Heart Failure,” published by the Japan Society of Hospital Pharmacists in April 2021. However, it should be noted that, in Japan, oxycodone is indicated only for cancer pain and is not applicable to patients with heart failure. Introducing opioids in patients with end-stage heart failure experiencing dyspnea even after adequate heart failure treatment should be considered. As demonstrated in this study, there were cases where dyspnea significantly improved and oxycodone was withdrawn; therefore, it is necessary to consider its introduction at an earlier stage in the future. Because of the retrospective study design, we could not determine the optimal dose and timing of oxycodone administration in our survey, and these issues should be further investigated in future studies.

### Efficacy of oxycodone for refractory dyspnea in patients with end-stage heart failure

Opioid treatment has been reported to be effective in the treatment of dyspnea due to cancer and/or non-cancer diseases such as chronic obstructive pulmonary disease [20, 21]. Oxycodone has also been reported to be as effective as morphine in the treatment of dyspnea in patients with terminal cancer [16, 17]. Although the mechanism of action of morphine and other opioids in improving dyspnea has not been fully elucidated, decreased perception of the central nervous system and sensitivity of the medullary respiratory center to carbon dioxide have been reported [22]. However, in heart failure, the opioid group showed significantly improved symptoms compared with the placebo group, although symptoms did not disappear. Although the goal of opioid treatment for dyspnea is to alleviate symptoms, the symptoms of dyspnea in patients with heart failure are highly severe, and opioids may be useful in improving their quality of life. MBS, a measure of dyspnea, in hospitalized patients with end-stage heart failure is shown in Fig. 1. The MBS score significantly decreased, suggesting the efficacy of oxycodone in treating refractory dyspnea in patients with end-stage heart failure. However, the study’s sample size was small, and thus it did not adequately demonstrate the efficacy of dyspnea. Further studies, including high-quality, large-scale prospective cohort studies and possibly randomized controlled trials, are required to determine the efficacy of oxycodone treatment compared with morphine for refractory dyspnea in patients with end-stage heart failure.

### Safety of oxycodone in patients with end-stage heart failure

Adverse events, including respiratory depression, nausea, vomiting, constipation, and delirium, are often experienced by cancer patients receiving morphine treatment [23]. Although there are concerns that oxycodone may cause adverse effects similar to those of morphine, in this study, the adverse effects caused by oxycodone were adequately managed with dose adjustments and symptomatic treatments. In two patients in this study, midazolam was concomitantly administered after oxycodone initiation; however, no serious adverse effects, such as respiratory depression, were observed. Morphine undergoes glucuronidation in the liver, whereas oxycodone is primarily metabolized by the CYP enzymes 2D6 and 3A4. Because the beta-blockers bisoprolol and carvedilol are metabolized by CYP2D6 and CYP3A4, it was hypothesized that their concomitant use with oxycodone could potentiate the effects of beta-blockers and decrease blood pressure and heart rate. However, in this study, no effects on these parameters were observed because the doses were small. Oxycodone does not inhibit CYP2C9, the major metabolizing enzyme of warfarin; therefore, its effect on warfarin pharmacokinetics and action is expected to be minimal. However, in this study, a prolonged PT-INR was observed in patients treated with the combination of oxycodone and warfarin. In particular, Case 5, involving a patient who was started on oxycodone while receiving warfarin showed a prolonged PT-INR. Although serum albumin level fluctuations are considered to be a potential contributing factor, the serum albumin level in this case remained stable between 2.7 and 3.0 g/dL during the entire treatment period, suggesting that there were no significant fluctuations. Therefore, it was hypothesized that oxycodone may have enhanced the anticoagulant effects of warfarin. This observation aligns with the findings of Hosokawa et al., who reported that the anticoagulant effects of warfarin were significantly enhanced when co-administered with oxycodone in patients with cancer. Specifically, the PT-INR increased significantly, and the Warfarin Sensitivity Index also increased, indicating an enhanced anticoagulant effect despite a reduction in the warfarin dose [24]. Therefore, it is recommended that PT-INR be closely monitored when initiating oxycodone therapy in patients receiving warfarin to prevent potential complications from enhanced anticoagulant effects [25].

### Study limitations

This study has several limitations. First, this was a single-center retrospective study with a small sample size; therefore, it was subject to various biases inherent to the data. Second, this study was conducted at a single institution, which limits its generalizability. Third, we could not ascertain whether dyspnea was relieved by treatment with oxycodone alone because of the various simultaneous treatments for heart failure. Fourth, we retrospectively evaluated adverse events based on descriptions in medical records. The frequency of adverse events may have been falsely decreased owing to the failure to record all events in the medical records, even though there were unacceptable symptoms. Fifth, clear inclusion/exclusion criteria and protocols for oxycodone administration were not established in this study. Moreover, the frequency of symptom evaluation and titration of oxycodone administration generally depended on the attending medical staff and varied among patients during the study period.

## Conclusion

This case series suggests that oxycodone may be a feasible treatment for refractory dyspnea in patients with heart failure. Side effects were also sufficiently eliminated by symptomatic treatments or adjustments to the oxycodone dosage without any serious adverse effects, such as respiratory depression. Oxycodone may be an alternative treatment option for dyspnea due to end-stage heart failure, in which morphine must be avoided because of renal dysfunction. Nevertheless, further studies are warranted to evaluate the safety and efficacy of oxycodone treatment, and oxycodone administration should be discussed by a multidisciplinary team.

## Data Availability

Data used in this report will not be shared owing to the risk of identifying the individuals.

## Abbreviations

MBS: Modified Borg Scale
eGFR: Estimated glomerular filtration rate
ARB: Angiotensin receptor blocker
ACE: Angiotensin-converting enzyme
PT-INR: International normalized ratio of prothrombin time
